# Evidence review and clinical guidance for the use of ziprasidone in Canada

**DOI:** 10.1186/1744-859X-12-1

**Published:** 2013-01-24

**Authors:** David M Gardner, Andrea L Murphy, Stan Kutcher, Serge Beaulieu, Carlo Carandang, Alain Labelle, Pierre Lalonde, Ashok Malla, Heather Milliken, Claire O’Donovan, Ayal Schaffer, Jorge Soni, Valerie H Taylor, Richard Williams

**Affiliations:** 1 Department of Psychiatry, Dalhousie University, Halifax, NS, Canada; 2 Department of Psychiatry, McGill University, Montreal, QC, Canada; 3 Department of Psychiatry, University of Ottawa, Ottawa, ON, Canada; 4 Centre de recherche Fernand-Seguin de l’Hôpital Louis-H. Lafontaine, Montreal, QC, Canada; 5 Department of Psychiatry, University of Toronto, Toronto, ON, Canada; 6 Department of Psychiatry, University of British Columbia, Vancouver, BC, Canada

**Keywords:** Ziprasidone, Expert consensus, Schizophrenia, Bipolar disorder, Depression, Anxiety, Dosing, Off-label

## Abstract

While indicated for schizophrenia and acute mania, ziprasidone’s evidence base and use in clinical practice extends beyond these regulatory approvals. We, an invited panel of experts led by a working group of 3, critically examined the evidence and our collective experience regarding the effectiveness, tolerability and safety of ziprasidone across its clinical uses. There was no opportunity for manufacturer input into the content of the review. As anticipated, ziprasidone was found to be effective for its indicated uses, although its utility in mania and mixed states lacked comparative data. Beyond these uses, the available data were either unimpressive or were lacking. An attractive characteristic is its neutral effect on weight thereby providing patients with a non-obesogenic long-term treatment option. Key challenges in practice include the need for dosing on a full stomach and managing its early onset adverse effect of restlessness. Addressing these issues are critical to its long-term success

## Introduction

Antipsychotic medications, including second generation agents (e.g. clozapine olanzapine, quetiapine, risperidone), have been prescribed more frequently, alone and in combination, and for an increasing breadth of indications in the last decade internationally [1-3]. These medications are used to treat schizophrenia and related psychotic disorders, as well as other conditions including mood, anxiety, and behaviour disorders. With the availability of a variety of antipsychotics each with a unique mix of advantages and disadvantages, clinicians and patients navigate treatment choices to identify preferred options. How do clinicians and patients make these decisions? Choosing an antipsychotic can depend on multiple complex factors. Following treatment selection, the immediate and real challenge of working towards achieving agreed upon goals of therapy begins (e.g., reduction in symptoms, relapse prevention, recovery).

The plan for this paper was to update previous work [4] and also introduce new information from a consensus panel of Canadian experts who met to develop a set of informed, clinically relevant suggestions for clinicians on the appropriate use of ziprasidone [4]. The original paper focused on schizophrenia and related psychotic disorders. This paper expands the focus to include bipolar disorder, major depression, and use in special populations. Adverse effects are extensively reviewed along with dosing and switching issues.

## Methods

A panel of twelve psychiatrists and two pharmacists with relevant antipsychotic related research and clinical expertise were convened on April 16, 2010 in order to discuss and synthesize suggestions for the clinical use of ziprasidone based on current research and clinical experiences. Funding was obtained as an unrestricted grant from Pfizer Canada’s community investment funding opportunity [5]. DG and SK served as the co-chairs for the meeting and AM led the development of the manuscript. At no time did the manufacturer or any of its representatives have any input into the manuscript. There were no representatives present during the meeting proceedings and they were not privy to the content of any version of the manuscript. Psychiatrists involved in the original publication were invited to attend by the co-chairs though not all were able to participate. New panel members, also invited by the co-chairs, with relevant clinical and research expertise were added to the panel based on pre-identified areas of interest and referrals from other panel members. Members were delegated a specific clinical area to critically analyze and present at the meeting. As well, approximately two hours were allotted and used to discuss time-intensive topics that were not sufficiently addressed by individual presentations. Prior to the start of the presentations, participants were asked to record in writing and verbally state to the group any potential conflicts of interest related to the pharmaceutical industry (e.g., grants, honoraria, advisory boards, investments, etc.) during the previous two years. During the development of this manuscript, all members were provided with two opportunities for critical and constructive feedback. This manuscript represents a synthesis of information from individual presentations, collective discussions, an extensive examination of the evidence base, and feedback from all panel members.

The following specific and general topics related to ziprasidone were sent to participants as requirements to be presented and discussed at the meeting: pharmacology, schizophrenia, early psychosis, bipolar disorder, major depression, adverse effects, cardiometabolic risk, children and adolescents, elderly, dosing by indication, and switching. Instructions were provided that presentations should cover relevant and critically appraised literature and include presenters’ clinical insights on their specific topic. Participants were offered assistance with conducting literature searches for their content area by the project leaders DG and AM and a research associate. When assistance with literature searches was requested, a comprehensive search of PubMed, Embase, ClinicalTrials.gov, Web of Science, the Cochrane Library, and a related references search of individual articles as well as review of references within papers was performed. Appropriate MeSH and text words were chosen based on the topic and no limits were applied other than to specific age groups (e.g., elderly) when appropriate.

## Results

### Pharmacology

Ziprasidone exhibits high binding affinity (inhibitory constants shown as pK_i_ with lower numbers indicating higher affinity) for select dopamine (D_2_: 3.1 nM; D_3_: 7.2 nM), serotonin (5-HT_2A_: 0.39 nM; 5-HT_2C_: 0.72 nM; 5-HT_1A_: 2.5 nM; 5-HT_1B/1D_: 2.0 nM; 5-HT_7_: 9.3 nM), and adrenergic (α_1_: 13.0 nM) receptors and moderate affinity for the histaminic H_1_ (47 nM) and dopamine D_4_ (32 nM) receptors. It distinguishes itself from other antipsychotics with its agonist action at the 5-HT_1A_ receptor and balanced reuptake inhibition actions at serotonin (53 nM) and noradrenaline (48 nM) transporters [6-8]. Refer to Table 1 for receptor binding affinities among commonly used antipsychotics.

**Table 1:** **Antipsychotic affinities (*K***_***i***_**) and target daily dosing range in schizophrenia**[7,9-11]

**Antipsychotic**	**Very high**	**High**	**Moderate**	**Low**	**Negligible**
daily dose	*(K*_*i*_ < 1.0)	*(K*_*i*_ 1.0–9.9)	*(K*_*i*_ 10–99)	*(K*_*i*_ 100–999)	*(K*_*i*_ ≥1000)
Ziprasidone	5HT_2a (0.4)_	5HT_1a (2.5) **_	α_1 (13)_	D_1 (130)_	M_1 (5100)_
120-160 mg	5HT_2c (0.7)_	D_2 (3.1)_	D_4 (32)_	α_2 (310)_	
		D_3 (7.2)_	H_1 (47)_		
		5HT_7 (9.3)_	NA_uptake (48)_		
			5HT_uptake (53)_		
			5HT_6 (76)_		
Aripiprazole	D_2__(0.3) **_	5HT_1a (1.7) **_	D_4 (44)_		M_1 (>1000)_
15-30 mg	D_3__(0.8)_	5HT_2a__(3.4)_	5HT_2c (15)_		
			5HT_7 (39)_		
			α_1 (57)_		
			H_1 (61)_		
			5HT_uptake (98)_		
Risperidone	5HT_2a__(0.3)_	α_1 (1.4)_	5HT_2c (10)_	5HT_1A__(210)_	5HT_uptake (1400)_
4-6 mg		D_2 (2.2)_	H_1 (19)_	D_1__(580)_	5HT_6 (2000)_
		5HT_7 (3.0)_			M_1 (2800)_
		D_3 (9.6)_			NA_uptake (28,000)_
		D_4 (8.5)_			
		α_2 (5.1)_			
Paliperidone		5HT_2a__(1.0)_	α_2 (17)_	5HT_1A__(590)_	M_1 (3570)_
6-9 mg		5HT_7 (1.3)_	H_1 (32)_	D_1__(670)_	
		α_1 (4.0)_	D_4 (30)_		
		D_2 (4.8)_	5HT_2c (71)_		
		D_3 (6.9)_			
Olanzapine		H_1__(2.8)_	5HT_2c (10)_	α_2 (170)_	5HT_1a (2100)_
10-20 mg		5HT_2a__(3.3)_	5HT_6 (10)_	5HT_7 (250)_	NA_uptake (2000)_
		M_1__(4.7)_	D_2 (20)_		5HT_uptake (>15,000)_
			D_3 (45)_		
			D_1 (52)_		
			α_1 (54)_		
			D_4 (60)_		
Clozapine		H_1__(1.8)_	5HT_6 (11)_	D_4 (54)_	5HT_uptake (3900)_
200-500 mg		M_1__(1.8)_	5HT_2c (17)_	D_2 (130)_	
		α_1 (4.0)_	α_2 (33)_	5HT_1a (140) **_	
		5HT_2a (8.9)_	5HT_7 (66)_	D_3 (240)_	
				D_1 (290)_	
				NA_uptake (390) *_	
Quetiapine		H_1 (8.7)_	α_1 (15)_	M_1 (100)_	α_2 (1000)_
400-800 mg				D_2 (180)_	D_1 (1300)_
				5HT_2a (220)_	5HT_2c (1400)_
				5HT_1a (230) **_	5HT_7 (1800)_
				D_3 (320)_	D_4 (2200)_
				NA_uptake (680)*_	5HT_6 (4100)_
					5HT_uptake (>18,000)_

* Quetiapine’s active metabolite N-desalkyl-quetiapine inhibits NA uptake (35 nM). Clozapine’s active metabolite N-desmethyl-clozapine also has a low affinity for NA uptake inhibition.

** Partial agonist. Quetiapine’s active metabolite N-desalkyl-quetiapine is a 5HT_1a_ partial agonist (191 nM). Clozapine’s active metabolite N-desmethyl-clozapine is a 5HT_1a_ partial agonist (105 nM).

5-HT: serotonin; D: dopamine; H: histamine; M: muscarinic cholinergic; α: alpha-adrenergic.

Lower values (shown in parentheses) indicate higher affinities at receptors or greater inhibition of pre-synaptic neurotransmitter uptake. Antipsychotics used in relatively small daily doses are unlikely to have clinical effects at receptors for which they have low affinity, unlike antipsychotics used at high daily doses.

Several pharmacologic actions of ziprasidone have long been predicted to confer an antidepressant and anti-anxiety effect with chronic use. Serotonin and norepinephrine transporter inhibition is dose dependent with similar *in vitro* affinity as tricyclic antidepressants [7,8,12]. *In vivo*, the clinical significance of its monoaminergic reuptake inhibition may be limited by plasma protein binding or be clinically relevant only at higher than currently recommended daily dosages.

Activity at 5-HT_1A_ is similar to that of buspirone [13]. Its inverse agonist activity at 5-HT_2A_ receptors disinhibits dopamine neurotransmission in the nigrostriatal, tuberoinfundibular, and mesocortical pathways possibly explaining its reduced risk of parkinsonism and hyperprolactinemia [7,14]. This action also disinhibits dopamine release in the mesocortical pathway linking the brainstem and prefrontal cortex, an action that is putatively linked to a reduction of the negative and cognitive symptoms of schizophrenia [15].

Also predicting a low rate of movement problems, such as parkinsonism, dystonia, and dyskinesia, is its relatively high 5-HT_2A_/D_2_ receptor affinity ratio [16-18]. Antagonism of the 5-HT_2C_ receptors disinhibits both dopamine and norepinephrine neurons in the cortex. This activity is predictive of improvements in cognitive and affective abnormalities as well as excitement and possibly agitation [13,15,19,20]. 5-HT_2C_ antagonist activity has also been associated with weight gain in some models, for example the 5-HT_2C_ knockout mouse model of obesity [21]. However, the association between 5-HT_2C_ antagonist activity and weight gain among antipsychotics is weak [22].

Ziprasidone has a low affinity for histaminergic_1_ (H_1_), muscarinic_1_ (M_1_), and α_1_-noradrenergic receptors. Among the biogenic amine receptors, H_1_ antagonist activity is associated with sedation and a valid predictor of weight gain liability [22]. Low affinity for α_1_-adrenergic receptors predicts a lower likelihood of orthostatic hypotension and sedation and low affinity for M_1_ receptors predicts low rates of anticholinergic side effects including dry mouth, blurry vision, urinary retention, constipation, memory impairment, confusion, and delirium.

Using an imaging protocol, 60% or greater D_2_ dopamine receptor occupancy is generally predictive of antipsychotic activity. An *in vivo* PET study examining the affinity of ziprasidone for dopamine (D_2_) and serotonin (5-HT_2_) receptors observed that optimal D_2_ receptor occupancy occurs at the upper end (≥120 mg/day) of the recommended dosage range [23].

### Pharmacokinetics

Ziprasidone achieves peak concentration approximately 6–8 hours post ingestion and steady state concentration by the end of the second day of dosing. The typical half-life is 6–10 hours, though individual patient values have ranged between 3 and 18 hours [14]. It is extensively metabolized, two-thirds by aldehyde oxidase, of which there are no known clinically relevant inducers or inhibitors, and one-third by P450 oxidation (3A4 > > 1A2). Potent inducers and inhibitors of CYP 3A4 may result in clinically relevant changes in ziprasidone clearance.

**Figure 1:**
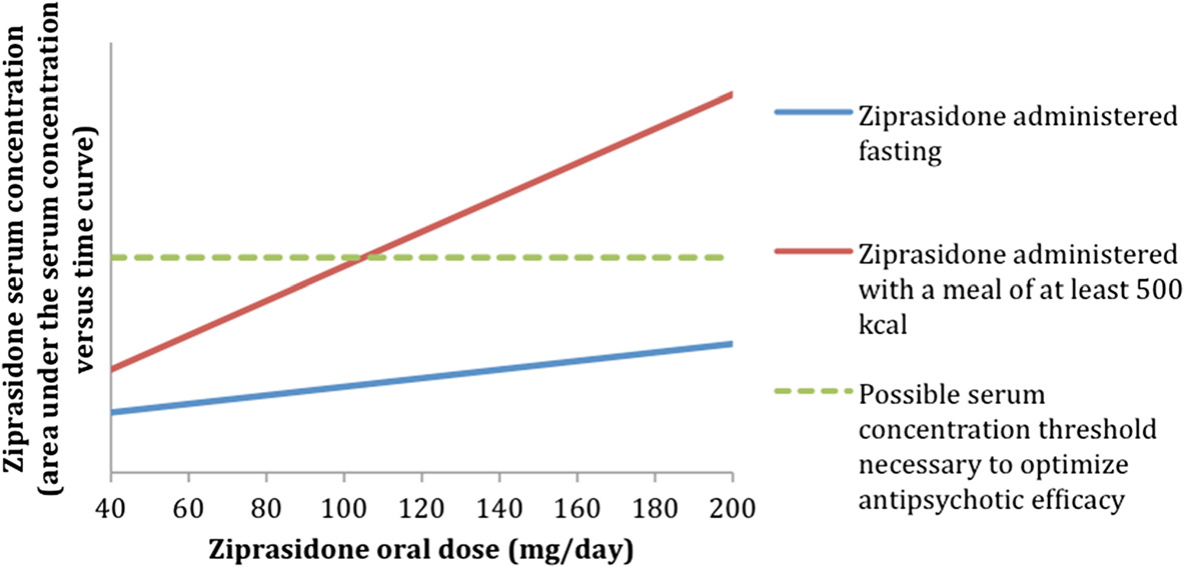
**Affect of food on ziprasidone absorption.** Reproduced with permission [25].

Unlike most antipsychotics, bioavailability of ziprasidone is approximately halved in the fasting state. To ensure optimal absorption, a meal of at least 500 kcal is recommended with each ziprasidone dose [24]. While absorption appears to be consistent whether the meal is low or high in protein or fat content, there are no data to indicate that simple carbohydrate meals (e.g., glucose-based energy drinks) are sufficient for absorption. It is important to note that dose and concentration are linearly correlated when taken with a ≥500 kcal meal but not in the fasting state. As such, doubling the dose in the fasting state does not achieve the same systemic bioavailability as taking the usual dose with food (see Figure 1). Table 2 provides calorie count examples of common foods.

**Table 2:** **Calories of common foods**[25]

**Item**	**Approx. kcal value**
1 large bagel	350
1 doughnut	120
1 cup of cornflakes cereal	100
Small fast food French fries	230
Ham and cheese sandwich	350
Hamburger, with bun, plain	270
Hotdog, with bun, plain	240
Egg sandwich in English muffin	300
Danish fruit pastry	335
1 egg, boiled	70
2 snack cakes with crème filling	250
1 slice pizza	350
20 potato chips (crisps)	200
1 orange	80
1 banana	90
1 apple	80

Reproduced with permission [25].

### Schizophrenia

The Canadian guidelines for the treatment of schizophrenia outline specific stages of therapeutic management [26]. These include individuals in the acute phase of illness requiring urgent care, first episode with little previous antipsychotic treatment, stabilization phase, stable phase, and those with multiple episodes. In addition, within these populations, subgroups such as those with persistent positive or negative symptoms, depression, suicidality, violent behaviours, inadequate response and gender differences are considered. Ziprasidone has been evaluated for many of these indications with analyses specific to some of the subgroups but details were not included in the 2005 iteration of the Canadian guidelines.

Evidence from ziprasidone randomized trials in patients with schizophrenia has been synthesized in a Cochrane Review [27]. The analysis included nine trials with the comparators amisulpride (not available in Canada), clozapine, olanzapine, quetiapine, and risperidone. In general, the quality and quantity of available data as well as extent of comparators was limited. Ziprasidone was determined to be less efficacious than olanzapine and risperidone based on PANSS total score and PANSS positive symptom subscore, especially in medium-to-long term studies [27]. Olanzapine was also associated with fewer hospitalizations [27]. One of the included trials comparing ziprasidone to clozapine in a head-to-head, randomized, double-blind study of 18 weeks in treatment refractory patients showed similar reductions in PANSS scores with ziprasidone and clozapine [28]. Further head-to-head trials are warranted to replicate these unexpected findings. Studies published since the review by Komossa et al., are primarily non-randomized or non-double blind designs [29,30] and several extension studies have also been published [31-33]. An extension study of 156 weeks following a 40 week randomized trial was designed to examine PANSS, quality of life scale, global assessment of functioning (GAF), and remission rates [33]. Treatment arms included ziprasidone 80–160 mg/d administered twice daily, ziprasidone 80–120 mg/d given once daily, and haloperidol 5 – 20 mg/d. Two hundred and twenty subjects of 599 were offered participation in the extension study and of these 186 consented to participate. In the 156 week extension study, mean PANSS negative subscale change scores per week based on last-observation-carried-forward (LOCF) were −4.86 (SD 7.64) for ziprasidone 80–160 mg, -3.17 (SD 6.15) for haloperidol 5–20 mg, and −2.87 (SD 6.48) for ziprasidone 80–120 mg. The least squares mean results of ziprasidone 80–160 mg/d compared to haloperidol were statistically different but not for haloperidol compared to ziprasidone 80–120 mg/d. GAF scores for either dosage group of ziprasidone were not different from haloperidol. In the post-hoc analyses, the rate of remission as defined by a score of less than or equal to 3 for at least 6 months on eight PANSS items was 51% in ziprasidone 80–160 mg/d group and 40% in the haloperidol group. No differences existed for the lower dose of ziprasidone compared to haloperidol. In another post hoc analyses of the same trial, Stahl et al., [34] explored aspects of negative symptoms and functioning. Kaplan-Meier Survival curves indicated greater rates of remission of negative symptoms with both dosage groups of ziprasidone versus haloperidol. At 1 year these rates were approximately 60% and 50%, and 35% for the higher and lower dose ziprasidone strategies and 35% with haloperidol. Another year later ~15% more patients in each group attained remission from negative symptoms. The findings suggest an advantage for ziprasidone for negative symptoms and indicate the extended duration of treatment required to observe substantial improvement in negative symptoms. Findings also indicated advantage for ziprasidone on 2 of 4 subscales (role functioning and community participation) of the Quality of Life Scale (QLS).

### Early psychosis

Results in first episode psychosis patients have been published across several papers based on the European First Episode Schizophrenia Trial (EUFEST) [29,35-38]. EUFEST was an open-label, randomized comparison of haloperidol, amisulpride, olanzapine, quetiapine and ziprasidone with a primary outcome of treatment discontinuation. Response rates, defined by Boter et al., [35] as symptom reduction of ≥50%, at 12 months were haloperidol 37%, amisulpride 67%, olanzapine 67%, quetiapine 46%, and ziprasidone 56%. Remissions lasting at least 6 months were observed at the following rates: haloperidol 17%, amisulpride 40%, olanzapine 41%, quetiapine 24%, and ziprasidone 28% [35].

Davidson et al., [37] reported moderate cognitive improvements with antipsychotics from the EUFEST data with no significant differences among agents [37,39]. Despite the younger patient population and use of a different first generation antipsychotic comparator, these findings are not dissimilar from the Clinical Antipsychotic Trials of Intervention Effectiveness (CATIE) results [39,40].

From available data for schizophrenia and first episode psychosis, aspects of the safety and effectiveness of second generation antipsychotics versus each other and their first generation counterparts are debatable [27,41,42]. It appears from the literature and panel discussions that ziprasidone’s place in therapy is best considered for patients at risk for cardiometabolic effects, those susceptible to sedation, and as a potential first line agent in those with first episode psychosis.

### Bipolar disorder

Second-generation antipsychotic studies in patients with bipolar disorder [43-46] typically show more benefits in patients with mania versus depression. Study results for second generation antipsychotics in bipolar depression have been mixed with quetiapine alone and olanzapine in combination with fluoxetine providing the most compelling data [47-51]. The World Federation of Societies of Biological Psychiatry (WFSBP) guidelines for bipolar depression indicate that the evidence for ziprasidone is negative [46]. The recent summary of the negative findings of two randomized, 6 week trials of ziprasidone monotherapy for people with bipolar depression sheds light on this issue. Lombardo et al. presented the results of both the fixed dose (40–80 or 120–160 mg/day), placebo-controlled study and the flexible-dose (40–160 mg/day), placebo controlled study [52-54]. Placebo response rates were 49% & 51% per study and ranged between 46% and 53% with ziprasidone.

Both studies were negative. Similarly, a ziprasidone augmentation study in bipolar I depression failed to find a benefit over lithium, divalproex, and lamotrigine monotherapies [55].

Ziprasidone was superior to placebo in a randomized trial of patients presenting with depressed mixed states (i.e., major depressive episode with 2 or 3 manic criteria symptoms). In an exploratory analysis ziprasidone was found to be more effective in the bipolar sample compared to the MDD sample (p = 0.036) [56]. Given the planned inclusion of depressive mixed state in DSM V, this study may prove to be of interest conceptually.

For patients with mania, some antipsychotics have been studied more extensively than others. For example, olanzapine has been supported as effective for not only the treatment of mania but also as maintenance therapy for the prevention of manic relapses [57,58] and may also demonstrate cost-effectiveness [57]. A systematic review and meta-analysis of randomized controlled trials of second-generation antipsychotics in the treatment of acute mania (including publications to 2006) is available [59]. Yildiz et al. [60] also conducted a meta-analysis of many agents, including ziprasidone, with purported antimanic effects. The Canadian Network for Mood and Anxiety Treatments (CANMAT) lists ziprasidone as one of twelve first-line treatment options for acute mania and as an option as an adjunctive therapy in maintenance of bipolar disorder [61]. The World Federation of Societies of Biological Psychiatry guideline for treatment of acute mania [45] support the use of this agent based on their definition of “A” level evidence, which primarily comes from three trials [62-64] with further information for recommendations presented based on cardiovascular safety considerations and restrictions on use. Pooled estimates [59] derived from Keck et al. (2003), [62] and Potkin et al. (2005), [63] that serve as the basis for the majority of supportive recommendations, demonstrate a standardized mean difference in YMRS scores of −0.44 (95% CI −0.65 to −0.23). This effect size estimate indicates that 67% of the ziprasidone group did better in terms of mania symptom reduction than the average placebo patient improvement [64] This was similar to individual pooled estimates for other individual second-generation antipsychotics (aripiprazole, olanzapine, quetiapine, and risperidone) as well as the combined pooled change (−0.45 (95% CI −0.57 to −0.32)) for the group of antipsychotic medications [55]. Yildiz et al., [60] meta analyzed data from Keck et al. (2003), [62] Potkin et al. (2005), [63], and Vieta et al. (2010), [65] and found a number needed to treat of 5.9 (95% CI 3.9 - 14.3) for mania management with ziprasidone compared to placebo. Few data for head-to-head comparisons are available with the exception of Vieta et al., [65] who found greater symptom reduction with haloperidol versus ziprasidone based on Mania Rating Scale (MRS) scores. Response rates at week 3 were: haloperidol 55%, ziprasidone 37%, and placebo 20%. Vieta et al. refer to the lack of equivalence between the agents’ doses (ziprasidone 116 mg/day vs. haloperidol 16 mg/day) [65] and others also comment in general on the relative lack of sufficiently long term trials of both placebo and head to head trials for informing decisions [60].

Pooled analyses of the trials by Keck and Potkin for subgroups of patients with a mixed manic episode and dysphoric mania are reported as positive trials [66,67]. However, in the dysphoric mania post-hoc analysis for example, baseline symptoms of depression were subsyndromal (HAM-D baseline ziprasidone 9.6 ± 4.6 and placebo 8.7 ± 4.1). The clinical relevance of the statistical difference in mean reduction in HAM-D scores is arguably negligible (ziprasidone −4.2, placebo −2.0). One study by Weisler et al. (2003), [68] is also cited in guidelines and other papers as supporting evidence but could only be found as an abstract.

Bowden et al. (2010), [69] conducted a 6 month, randomized double-blind trial of ziprasidone or placebo added to either lithium or valproate in patients with continued symptoms of bipolar I disorder (mania or mixed episode). Participants first received ziprasidone and lithium or valproate for an 8 week open label stabilization phase. Of the 584 participants that received ziprasidone add on therapy, 240 were then randomized to ziprasidone or placebo plus mood stabilizer. Approximately 67% and 80% of participants were relapse free in the placebo and ziprasidone groups at 6 months (p = 0.01). The relapse free data was primarily attributed to differences in manic episodes for ziprasidone as depressive episodes were similar between groups. Kaplan-Meier estimates indicated a significant difference (log rank test p = 0.0104) for time-to-intervention for a mood disorder favouring ziprasidone. The mean difference for ziprasidone versus placebo in the mania rating scale (MRS) total score was −3.27 (95% CI −4.91 to −1.62).

Potential advantages of ziprasidone in terms of metabolic-related side effects maintain interest in this medication for bipolar disorder. However, experiences of agitation and insomnia have limited its clinical utility in some manic and hypomanic patients. Concomitant benzodiazepine use in mania trials may have helped to limit these adverse outcomes.

### Depression

The evidence for the use of ziprasidone for the treatment of major depressive disorder (MDD) is sparse. The argument for evaluating its role for the treatment of MDD is made from several perspectives including plausible pharmacological hypotheses and inadequacy of currently available treatment options.

Other second-generation antipsychotics (e.g., olanzapine, risperidone, and quetiapine) have already made the transition from use for schizophrenia to use in mania and most recently to depression. Ziprasidone’s pharmacological actions, including its 5-HT_2A_ antagonism and reuptake inhibition of 5-HT and NE suggest an antidepressant effect. However, making explicit linkages between receptor binding profiles and clinical results in various patient groups is not straightforward. For instance, a sub-analysis of depression related outcomes in a meta-analyses of patients with schizophrenia demonstrated symptom benefits in those antipsychotics with (e.g., quetiapine) and without (e.g., olanzapine) 5-HT_1A_ partial agonistic effects [70].

It should be noted that, at present, there are no randomized, double-blind, controlled trials of ziprasidone in MDD published in peer reviewed journals. However, three monotherapy randomized trials have been completed (primary data collection ended June 2010 or earlier) and one ziprasidone augmentation of SSRI treatment study is expected to be completed in 2013 [71-74]. Methodological and recruitment details of each can be found at clinicaltrials.gov but no results are provided.

Other second generation agents have more information related to depression as acute therapy augmentation strategies. The CANMAT guidelines for the treatment of major depressive disorder include quetiapine as a second-line recommendation [71-74]. In those patients who have incomplete or non-response to an initial antidepressant, “first-line” approaches include add-on therapy with aripiprazole (Level 1), olanzapine (Level 1), and risperidone (Level 2). Ziprasidone is suggested as add-on therapy in the third line approach with level 3 evidence [75].

A meta analysis by Nelson and Papakostas (2009) synthesized information on the use of second generation agents as augmentation therapy in major depressive disorders [76]. The odds ratio for response, as defined by ≥ 50% reduction in MADRS or HAM-D score, with second generation antipsychotics was 1.69 (95% CI 1.46, 1.95). Odds for remission was doubled (OR = 2.00, 95% CI 1.69, 2.37). Second generation agents included in the review were aripiprazole, olanzapine, quetiapine, and risperidone. Methodological issues and the unresolved therapeutic questions of what is the appropriate dose and duration of second generation antipsychotics remain despite this meta-analysis. Some agents such as quetiapine have been studied further since the time of the search conducted by Nelson and Papakostas, with promising results [77]. Komossa et al. (2010), [78] also published a systematic review of second generation antipsychotics for major depressive disorder and dysthymia and noted the relative lack of available published data for several agents including ziprasidone. Two available studies [79,80] were excluded from their analyses as they were not RCTs [78].

Two open-label randomized ziprasidone studies in treatment-resistant depression have been published. Dunner et al. (2007), [81] allocated 54 patients to combination continued sertraline therapy or add-on ziprasidone 80 (Z80&S) or 160 mg/day (Z160&S). Mean change (SE) MADRS scores were −8.3 (2.2) for Z160&S, -6.0 (1.9) for Z80&S, and −4.4 (2.0) for sertraline alone. Possibly reflecting the small sample size, differences among groups were not significant. Differences in response (≥50% reduction in MADRS score) and remission (MADRS score < 10) rates of 6/19 (32%), 4/21 (19%), and 2/20 (10%), and 4/19 (21%), 1/21 (5%), and 1/20 (5%), respectively, were also not significant. Papakostas et al. (2004), [80] studied ziprasidone add-on therapy to SSRIs in a 6 week study with 20 patients, of which 13 completed the study. In the intention-to-treat analysis 10 of 20 participants achieved a 50% reduction in HAM-D-17 scores and were thus defined as responders. Other small and uncontrolled investigations [79,82] do not contribute significant information for therapeutic decision-making. The available pharmacological hypotheses and sparse data may warrant further exploration of ziprasidone in managing patients with MDD.

### Adverse events

The prevalence and severity of various adverse effects is heterogeneous amongst antipsychotics but potential consequences can be universal and include impairment in quality of life, poor adherence, stigmatization, morbidity and mortality [83]. Similar to other antipsychotics, the risk:benefit profile of ziprasidone requires careful consideration and panel participants discussed potential merits of this agent (e.g., metabolic adverse events) alongside drawbacks (e.g., agitation). Other detailed reviews on these topics have been published [84]. During the discussions regarding adverse events, the panel focused on several clinically concerning or potentially limiting adverse events with ziprasidone and with reference to other antipsychotics to provide context.

#### Agitation

The group discussed the potential for discontinuation and other deleterious effects on patient outcomes due to therapy-related agitation. From the group’s clinical experience and review of the literature, it was noted that manifestations of agitation are not well described and could be classified as illness symptoms (e.g., hypomania) or referred to as a different adverse effect (e.g., akathisia). Differences in terminology are also plausible with some references to activation [85], which may have considerable symptom overlap with manic or hypomanic symptoms, akathisia, and agitation. Patients with agitation can demonstrate a variety of behaviours (e.g., pressured or loud speech, pacing, tapping fingers or feet, starting, moaning, appearing distracted by an internal stimulus) and also manifest physiologic changes related to autonomic tone (e.g., blood pressure) and the musculoskeletal system (e.g., muscle tension) [86]. The group discussed the importance of ruling out and reversing potential contributory etiologies to the symptoms. As an example, patients with concurrent substance use problems may describe activation or appear agitated in relation to the substance dependence.

There is controversy as to whether the symptoms of agitation or activation are dose-dependent and whether any variability exists in terms of commencement of symptoms in relation to duration of therapy. Clinical experience within the group supported that agitation at lower doses is a phenomenon reported clinically by patients and suggestions were made regarding dose initiation and up-titration, as discussed later, to enhance the likelihood of adherence in early stages of treatment. Exemplifying the challenge of ziprasidone-related agitation, Kaushik (2009) [85] reported activation with higher, not lower, doses of ziprasidone, and reviews the related literature and putative mechanisms [15]. Clinicians should also be aware of reports of mania like symptoms associated with the use of ziprasidone when considering symptoms of activation and agitation [87].

#### QT_c_ prolongation

QT_c_ prolongation is a risk factor for serious ventricular dysrrhythmias (e.g., Torsades de pointes (TdP)) and sudden cardiac death. The frequency of TdP with non-cardiac classes of medications is not well defined but is thought to be well below 0.1% [88]. Harrigan et al. reported on the changes in QTc with 6 antipsychotics including ziprasidone [89]. The mean change in QTc (msec) was 15.9 with ziprasidone, which was approximately 9 to 14 msec more than other included agents (haloperidol, quetiapine, risperidone and olanzapine) but less than thioridazine (30.1 msec). Addition of ketoconazole, which served as a metabolic inhibitor in the study, did not appreciably increase the QTc any further with ziprasidone (16.6 msec) [89]. The authors also state that at no point did any participant experience a QTc greater than 500 msec, which is generally considered as approaching the threshold for TdP [89]. Miceli et al. (2010), [90] examined the effects of very high dose ziprasidone (up to 320 mg/day) and haloperidol on QTc. The mean QTc change was 19.5 msec (95% CI 15.5 to 23.4) for ziprasidone 160 mg/day and 22.5 msec (95% CI 15.7 to 29.4) at 320 mg/day. One patient of 29 experienced a change in QTc of 61 msec at the 320 mg/day dose. No patients in either treatment arm had a QTc of greater than 450 msec [90]. Eker et al. (2009) [91] reported on three cases of potential ziprasidone induced QTc prolongation with one patient experiencing a QTc of 510 msec with a dose of 240 mg/day. Resolution of QTc prolongation was achieved in all cases with dose reduction or cessation of ziprasidone. Clinical symptoms of prolonged QTc were not reported. Rocha et al. (2006), [92] reported one older nursing home patient with a QTc of 505 ms from a baseline of 450 ms. The per protocol group (n = 15) showed a 29 ms increase in mean QTc (baseline: 409.5 ± 15.5, day 49: 438.5 ± 10.6 ms).

In their meta analyses examining ziprasidone for schizophrenia, Komossa et al. [27] reported no apparent differences for prolonged QTc and changes in QTc from baseline with ziprasidone as compared to clozapine, amisulpride, olanzapine and risperidone. These findings are similar to those of Chung and Chua [93].

As of January 31, 2010 in Canada, Health Canada received nine voluntary, spontaneous reports of QT prolongation with ziprasidone [94]. This information is provided with the caveat that spontaneous reports have important limitations and are not appropriate for determining probability of adverse events.

Alipour et al. (2010) [95] reported a case of TdP in a ziprasidone overdose (6 g) with co-ingestants. They indicate that their case represents the second published overdose of ziprasidone resulting in TdP (the other case involved a hypokalemic 30 year old woman) and note that other overdose reports did not lead to adverse cardiac outcomes [95,96]. Tan et al. (2009) [97] conducted a systematic review in order to examine the literature for cardiovascular effects following second generation antipsychotic overdoses. In their review, 1 pediatric case of increased QTc in a child under 7 years of age receiving a ziprasidone dose of 400 mg was identified. From case level data in adults 16 years and older, increased QTc was reported in 13 cases, ventricular arrhythmia in one, and death in two. Two cases of QTc prolongation occurred while patients were taking both ziprasidone and quetiapine in one and ziprasidone and risperidone in the other. The deaths were polysubstance overdoses with one patient having prolonged QTc and ventricular dysrrhythmia with suspected co-ingestion of amantadine, ibuprofen and ziprasidone. Hypokalemia and bradycardia were features of the patient’s presentation.

Considerable discussion occurred with the panel regarding routine screening with electrocardiogram (ECG) and whether it is an effective way to detect individuals at risk for persistent prolonged QTc interval. ECG is recommended before initiating treatment with ziprasidone in patients with stable cardiac disease or if cardiac symptoms (e.g., syncope, palpitations, vertigo) occur during treatment. Clinicians should be knowledgeable of the contraindications listed in the product monograph and should also consider the many other conditions, population characteristics (e.g., older women), and medications that can affect parameters of the cardiovascular system including impulse generation, conduction, and hemodynamics. The group speculated that given the best available evidence, duration of use (since 2001 in the US and 2008 in Canada), and the potential breadth of use for off-label indications in patient types not usually reflected in clinical trials, if ziprasidone was truly associated with a risk of sudden death from cardiac causes its prevalence would be apparent from literature and clinical experience. However, concerns exist concomitantly regarding capturing post-marketing adverse reactions and the voluntary nature and low levels of reporting by health care professionals in the Canadian context. Concerns also exist for the relative low quantity of effectiveness and safety data in certain populations (e.g. pediatrics) and if off-label use was to occur in these groups, uncertainty exists as to the effects on QTc.

#### Extrapyramidal side effects

In the meta-analysis by Komossa et al. (2009), [27] various domains of extrapyramidal symptoms (EPS) were analyzed for ziprasidone versus other comparators (i.e. amisulpride, quetiapine, olanzapine and risperidone). There were no statistically significant differences in the rate of movement related side effects with ziprasidone versus amisulpride, quetiapine, and olanzapine. The difference in abnormal movements, akathisia severity, and parkinsonism was statistically significant in favour of ziprasidone compared to risperidone, a finding that comes from a single comparative study [98]. Based on two studies in the meta-analysis, the rates of antiparkinsonism medication use were 14.1% with ziprasidone versus 16.6% with risperidone, with a Mantel-Haenszel estimated risk ratio of 0.70 (95% CI 0.51, 0.97). The risk of using anti-parkinsonism medication was significantly increased with ziprasidone (16.8%) compared to olanzapine (10.9%) with a Mantel-Haenszel risk ratio of 1.43 (95% CI 1.03, 1.99). Compared to quetiapine, rates were 7.6% with ziprasidone compared to 3.3% with quetiapine with a Mantel-Haenszel risk ratio of 2.32 (95% CI 1.07, 5.00). Similar findings are published by Rummel-Kluge (2010) [99].

From the available literature, arriving at conclusions with respect to EPS risk remains difficult due to differences in study design, the perspective of the analyses, the patient population, and how adverse events were captured and reported. An analysis by the CATIE investigators found no significant differences in ratings for parkinsonism, dystonia, akathisia, or tardive dyskinesia when examining second-generation agents [100]. When considering EPS in the context of switching, as per the results of three 6-week open-label studies, potential improvements with Simpson-Angus scale scores after switching from conventional agents and risperidone to ziprasidone occurred but not when switching from olanzapine [101].

Regarding serious movement disorders (e.g., oculogyric crisis, acute and tardive dystonias, tardive dyskinesia, neuroleptic malignant syndrome), the panel discussed that from clinical experience the risk of severe EPS appears to be uncommon or rare with ziprasidone but can occur. The comparative risk of individual severe movement disorders is difficult to evaluate because of their indistinct reporting in clinical trials and overall infrequency [102-108].

#### Rash

Pre-marketing trial data represented in product monograph information from the US for ziprasidone indicates that about 5% of patients receiving ziprasidone developed rash and/or urticaria.^112^ Resolution of symptoms was reported with symptomatic management (e.g. steroids, antihistamines) and/or discontinuation of treatment [109]. As reported by the manufacturer, treatment emergent adverse reactions in short term trials of patients with schizophrenia demonstrated rash in 4% of ziprasidone patients and 3% of placebo recipients. In bipolar patients, treatment emergent pruritis was reported in 3.3% of ziprasidone patients and 2.2% of placebo recipients [14]. Post-marketing information from spontaneous reports as cited by the manufacturer includes the potential for angioedema and Stevens Johnson Syndrome although no causality with ziprasidone has been established [14]. Published cases describe potential ziprasidone-induced angioedema, urticaria and angioedema, and photoallergic skin reaction [110-112].

As with any cutaneous reaction, the suspected etiology and contributing factors requires in depth exploration and depending on the nature and severity, consultation with specialists (e.g., dermatologists, immunologists). For ziprasidone, it is suggested that the medication be discontinued and re-assessed when other compelling etiologies are absent and symptomatic treatment to optimize resolution should be pursued. Expedient consultation with specialists for advice and recommendations regarding ambiguous clinical situations of cutaneous reactions may be sought when pharmacotherapeutic alternatives to ziprasidone are limited due to individual patient characteristics and/or the risks associated with discontinuation of treatment are considered to be dire.

#### Cardiometabolic risks

Given the concern for potential disparities in the quality of health care services for patients with mental health disorders and the higher odds of adverse health related outcomes including mortality and morbidity, consideration of therapeutic alternatives that minimize risks is essential. Differences among antipsychotics are frequently reported in studies for outcomes that are considered clinical and/or surrogate in nature (e.g., weight gain, cholesterol, glucose, etc.) and therefore these risks must be weighted with the individual patient profile and benefits.

#### Weight gain

The ideal measure or best discriminator (e.g., BMI, abdominal waist circumference, etc.) for predicting the risk of cardiovascular outcomes with respect to adiposity remains controversial [113,114]. The explicit mechanism of antipsychotic induced weight gain is not known but various theories and multifactorial explanations have been and continue to be explored including significant interest in genetics [115-122]. Foley and Morley report lower pre-treatment BMI, younger age, triglyceride levels, more negative symptoms, antidepressants, and more concomitant medications were predictive of weight gain following antipsychotics initiation in first episode psychosis patients [113]. Their findings are consistent with other reviews [123]. Some researchers and clinicians have also speculated that symptom improvement for some patients may be correlated with weight gain although currently available research does not support clinically important improvements in PANSS and CGI with weight gain for patients with schizophrenia [124].

Weight gain liability varies among the commonly used antipsychotics. For example, compared to amisulpride, olanzapine, quetiapine, and risperidone, respectively, the RR of a weight gain of ≥7% of baseline body weight with ziprasidone was 0.48 (95% CI 0.18, 1.29), 0.22 (95% CI 0.14, 0.33), 0.45 (95% CI 0.28, 0.74), and 0.49 (95% CI 0.33, 0.74), respectively, identifying a distinct clinical advantage of ziprasidone therapy [27]. Mean weight change (kg) favoured ziprasidone compared to olanzapine −3.82 (95% CI −4.69, -2.96), quetiapine −1.20 (95% CI −2.45, 0.05), and risperidone −1.10 (95% CI −2.35, 0.15) [27]. The systematic review by Rummel-Kluge (2010) similarly found a weight management advantage with ziprasidone compared to olanzapine −3.82 kg (95% CI −4.69 to −2.96), quetiapine −1.2 (95% CI −2.45 to 0.05), and risperidone −1.1 kg (95% CI −2.35 to 0.15) [125]. In a database analyses, Parsons et al. (2009) [126] examined data of short and long term placebo and active controlled trials of various antipsychotics. The long-term trials for the antipsychotics studied (i.e., haloperidol, olanzapine, risperidone, ziprasidone, and placebo) included 1649 patients with 450 patients treated for up to 6 months and 470 patients with potential exposure for up to one year. For ziprasidone long-term trial data (N = 1080), the random effects model for a weight gain of greater than 7% was 12% (95% CI 8%, 16%) and for weight loss greater than 7% was 16% (95% CI 11%, 21%). Mean weight change per month was a mean of 0.37 pounds (95% CI −0.83, -0.09) and the median loss was 0.17 pounds [126]. In the CATIE trial (2005) [127], weight gain of ≥7% from baseline to last observation occurred in 30% of olanzapine, 16% of quetiapine, 14% of risperidone, 12% of perphenazine, and 7% of ziprasidone patients.

Weiden et al. (2008) [128] analyzed data from participants (N = 185) in three ziprasidone pre-marketing (between 1997 and 1999) extension studies that were originally 6 weeks in duration and could be extended to a maximum of 58 weeks. In the first phase of the study patients were switched from risperidone (N = 43), olanzapine (N = 71) and conventional antipsychotics (N = 71) to ziprasidone. In the extension phase, 61% of participants discontinued treatment by 58 weeks with 54% of those agreeing to participate in the extension study completing 32 weeks of treatment. Last observation carried forward and observed case was used for the analyses. Patients switched from olanzapine and risperidone lost 4.5 kg and 5.1 kg, respectively (p < 0.01 for both). There was no statistical difference in weight loss in those switched from conventional antipsychotics.

Switching to ziprasidone was discussed in the context of seeking improvement in weight and other metabolic parameters, however the panel noted that these potential benefits must be weighed in light of effectiveness and the chance of successful treatment with sequential agents [101,129-131].

#### Cholesterol and glucose

From meta-analytic data of head-to-head comparisons of second generation agents in schizophrenia [125], ziprasidone had a favourable profile as compared to olanzapine (−15.83, 95% CI −25.72 to −5.95), quetiapine (−16.01, 95%CI −23.46 to −8.57) and risperidone (−8.58, 95% CI −16.04 to −1.11) for mean difference in cholesterol (mg/dL). Ziprasidone was significantly different in terms of mean difference in glucose (mg/dL) as compared to olanzapine (−8.25 -95% CI −13.72 to −2.77) but not significantly different from quetiapine and risperidone. Weiden et al. (2008) [128] also demonstrated improvements in total cholesterol and triglycerides with the use of ziprasidone as compared to olanzapine and risperidone.

The link between changes in lipids and glycemic control and adverse macrovascular and microvascular complications varies among pharmacotherapies, with some drugs known to cause beneficial effects on surrogate measures but poor cardiovascular outcomes. As such, the implications of the variances in negative effects of antipsychotics on surrogate markers of cardiovascular disease should not be assumed. Weinmann et al. (2009) [132] conducted a systematic review examining the available literature on antipsychotic-related mortality, including cardiovascular morbidity and mortality, and found inconsistent findings. The authors also comment that the long term effects of second generation antipsychotics will require longer term follow-up.

Significant attention and research has also been devoted to finding interventions to attenuate or mitigate antipsychotic induced weight gain and related parameters (e.g., HbA1c, lipids) [115,126,133-138]. For example, a recent review by Maayan et al. examined 32 studies with 15 different pharmacological agents as potential treatments for antipsychotic associated weight gain [133]. The review revealed an absence of head-to-head trials for pharmacological options and mixed results with regard to achieving and sustaining weight reduction benefits possibly accounted for, in part, by differences in timing of initiation of therapy. As a result, the panel discussed a need for additionally focusing efforts on prevention and health promotion. Continued study regarding morbidity and mortality trends and the etiology of these outcomes for those with symptoms of mental illnesses and disorders is required. Implementation of evidence-informed standards of care and recommendations for screening, prevention and monitoring for physical, including cardiometabolic risks, [139] and mental health care are required.

### Ziprasidone use in young and older populations

#### Children and young adults

The use of second generation antipsychotics in the pediatric population is controversial and, as accurately observed by Vitiello et al. [140], practice has expanded more rapidly than the evidence base. The primary concerns in this population are overuse, off-label use, and lack of effectiveness and safety data for specific patient groups and indications [140-160]. Concerns regarding the long-term safety of these agents require specific investigation to support informed decisions. For example, the cardiometabolic parameters (e.g., weight gain, cholesterol) potentially influenced by several second generation antipsychotics and the impact of these changes on health outcomes may not be realized for a considerable time following exposure [161].

Notably, the second generation antipsychotics are not approved by Health Canada for use in pediatrics, and in the US ziprasidone is not indicated as a first-line therapy in pediatric populations. Despite the absence of regulatory approvals of antipsychotics in pediatrics internationally (with few exceptions), these medications are used commonly in pediatrics. A recent study of US Medicaid recipients in Michigan identified 292 individuals under 21 years of age receiving ziprasidone prescriptions in the year following availability on the market in the US. The majority (58%) of recipients were between 12 to 17 years. The data also demonstrated that in 33% of children, ziprasidone was their first antipsychotic prescription and of the beneficiaries receiving ziprasidone 53% had a diagnosis of psychosis [162]. Thirty six percent of prescriptions were written by general practitioners. These findings suggest a general willingness to prescribe an untested psychotropic in a vulnerable patient population.

The use of ziprasidone in the child and adolescent population was reviewed recently by Elbe and Carandang (2008) [163]. An updated search did not produce additional relevant information on effectiveness. In clinical practice ziprasidone would be considered a second-line treatment for Tourette’s or other tic disorders, bipolar disorder, and for autism spectrum disorders, [164,165] and it may be considered in patients in which weight gain or metabolic adverse effects to other standard antipsychotics are a particular concern [161,166].

The use of this medication should be weighed in context of the available evidence for efficacy and safety in pediatrics. To date, two published prospective, double-blind RCTs, with a combined total of 266 children, are available that examined ziprasidone for Tourette’s syndrome [167] and bipolar mania, schizophrenia, or schizoaffective disorder [168]. The remaining evidence of effectiveness and safety consists of open-label, non-randomized trials, with a total of 428 participants. From the 2008 review by Elbe and Carandang, [163] other published evidence in the form of retrospective data or case series contributes information from approximately 225 patients.

From a safety and tolerability perspective, it appears that sedation and somnolence are the most common adverse events reported with ziprasidone in pediatric populations [169]. Reports of serious adverse effects involving ziprasidone have included neuroleptic malignant syndrome [106,170], EPS [171,172], and changes in cardiac rate or rhythm including QT_c_ prolongation [173,174]. Although published case reports are available, [175,176] accurate estimates of adverse effect rates are lacking. Difficulty remains with establishing recommendations for monitoring for some side effects such as QTc given the finite amount of data available from studies and post-marketing surveillance. Until more safety data is available we recommend an ECG at baseline and at the target dose when used in children and youth.

#### Older adults

Despite the warnings of increased risk of death [109] with the use of second-generation antipsychotics in older adults with dementia, there continues to be substantial off-label use in this population. There are no randomized controlled trials evaluating the efficacy or safety of ziprasidone in elderly patients [177]. A Cochrane Review examining atypical antipsychotics for aggression and psychosis in patients with dementia does not include ziprasidone [178]. One open-label, flexible dosing prospective case series of 25 nursing home patients with dementia, of which 15 completed the study according to protocol, by Rocha et al. (2006) [92] showed a significant reduction in the mean total neuropsychiatric inventory (NPI) score from baseline 47.1 ± 17.1 (mean ± SD) to 25.8 ± 17.9 at 49 days. From clinical experiences, appropriate management of agitation, aggression, and confusion, remains complex with few safe and effective therapeutic alternatives.

### Long-term treatment acceptability

Persistence with prescribed treatment is a critical goal of therapy with antipsychotics ^182,183^ and yet it remains overestimated in the clinical setting despite ample characterization of non-adherence in the literature [179-183]. Kamossa et al. (2009), [27] reviewed ziprasidone versus other atypical agents for schizophrenia and found premature discontinuation for any reason in 59% of people randomized to antipsychotic treatment, a finding matched by others [184]. In the review by Komossa, the relative risk of leaving studies for any reason was more likely with ziprasidone as compared to olanzapine, 1.26 (95% CI 1.18, 1.35), and risperidone, 1.11 (95% CI 1.02, 1.20) [27]. The CATIE investigators demonstrated all cause discontinuation rates of 64%, 74%, 75%, 79%, and 82% for olanzapine, risperidone, perphenazine, ziprasidone, and quetiapine, respectively [127]. A recent naturalistic study in patients with symptoms of acute psychosis demonstrated discontinuation rates of approximately 60% for the second generation antipsychotics (quetiapine, olanzapine, risperidone and ziprasidone) within 100 days [181]. Kahn et al., examined the effectiveness of antipsychotic drugs in first-episode schizophrenia and schizophreniform disorder and had 12 month discontinuation rates (any cause) of 72% (haloperidol), 53% (quetiapine), 45% (ziprasidone), 40% (amisulpride), and 33% (olanzapine) [29]. A study by Olfson et al. (2011) [160] showed discontinuation rates in the first 180 days with early onset schizophrenia to be above 70% for all agents including aripiprazole, olanzapine, quetiapine, risperidone, and ziprasidone. Many other trials with ziprasidone in patients with schizophrenia or related disorders that were small or large in scale and duration have shown similarly significant discontinuation rates regardless of their design (i.e., prospective or retrospective) [30,31,33,185,186].

Rates of discontinuation are similarly high for other mental disorders. Randomized studies in patients with bipolar disorder demonstrate completion rates of 54% and 61% for ziprasidone versus 85% and 44% for placebo [58,59]. Vieta et al. showed completion rates of 45% for haloperidol, 41% for ziprasidone and 28% for placebo in patients with acute mania in a 12 week study [60]. In patients with treatment resistant depression who were randomized to either open-label sertraline monotherapy or sertraline and ziprasidone (80 or 160 mg), 75%, 50% and 47% of participants completed the study, respectively [81]. A meta-analysis of second generation agents, not including ziprasidone, for augmentation therapy in major depressive disorder demonstrated an odds ratio of 1.30 (95% CI 1.09, 1.57) for discontinuation for any reason [75].

### Dosing and switching challenges and solutions

#### Dosing

Evidence informed clinical experience suggests that optimal dosing of ziprasidone in first episode patients appears to be in the range of 80–160 mg/d, for chronic schizophrenia 120–160 mg/d, for bipolar mania 120–160 mg/d. While evidence of benefit in major depression is lacking, when tried, a lower dose of 20–80 mg/d is recommended.

Dosing should be started at 40 mg twice daily with meals for inpatients and 20 to 40 mg twice daily for outpatients. For example, a suggested schedule for an early psychosis outpatient is: Day 1: 20 mg AM, 40 mg PM; Day 2: 40 mg AM, 60 mg PM, Day 3: 60 mg AM, 60 mg PM, then reassess. The second dose of the day should be taken with the last meal of the day, and patients should be informed about the importance of consuming 500 kcal of food with each dose of their ziprasidone.

**Figure 2:**
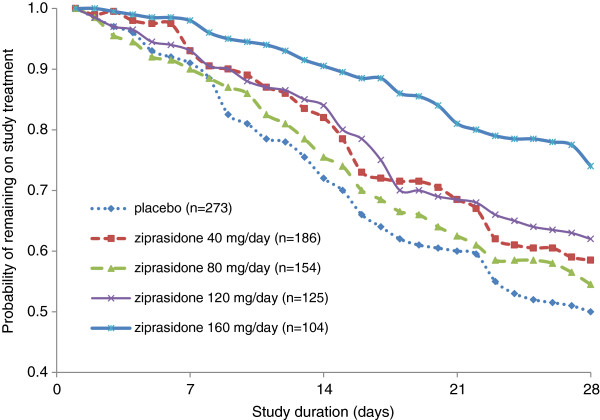
**All-cause discontinuation survival curves by ziprasidone dose.** Reproduced with permission [185].

The traditional approach to dosing antipsychotic medications is to start at a low dose and gradually titrate upward based on patient tolerance and response. This approach has been challenged of late with findings that a more rapid titration, for example with quetiapine, may increase the chance for successful long-term therapy [187,188]. This may also apply to ziprasidone [185,189]. A pooled re-analysis of discontinuation rates at or before 4 weeks of treatment was conducted based on 4 placebo-controlled, fixed-dose studies (n = 842) of ziprasidone. The rate of all cause discontinuation was lowest with ziprasidone at its highest dose, primarily driven by its greater efficacy. The respective rates of discontinuations for all reasons, lack of efficacy, and adverse effects by dose were: placebo: 50%, 31%, 2%; 40 mg/d: 41%, 24%, 2%; 80 mg/d: 46%, 22%, 2%; 120 mg/d: 38%, 18%, 7%; and 160 mg/d: 27%, 11%, 4%. Figure 2 illustrates greater treatment persistence is detectable as early as 7 to 14 days when the higher target dose of 160 mg/d is prescribed.

Ziprasidone dosing recommendations vary depending on the diagnosis and age of the patient and should also be sensitive to patient-specific safety, tolerability and efficacy issues inferred from past treatment response and medical considerations [9,109]. For dose guidance refer to Table 3.

**Table 3:** Ziprasidone dosing recommendations*[9,109,163]

	**Start dose & titration rate**	**Target dose range**	**Maximum dose**
Schizophrenia (adults)	20-40 mg twice daily	60-80 mg twice daily	100 mg twice daily
	↑ every 1–3 days		
Bipolar mania (adults)	40 mg twice daily	60-80 mg twice daily	80 mg twice daily
	↑ every 1–2 days		
Elderly	No differences in the pharmacokinetics of ziprasidone were observed in a small study involving healthy elderly patients. A limited number of elderly with schizophrenia were exposed to ziprasidone in clinical trials who demonstrated similar tolerability as adults. These findings suggest that reduced dosing may not be necessary unless other considerations are present. Ziprasidone, like other atypical antipsychotics, is not indicated for elderly patients with dementia due the elevated risk of death.		
Children	There is limited experience with ziprasidone in children under clinical trial conditions, as such data to support its safety and efficacy at any dose is insufficient. Mean dose is one small RCT in children (11.5 y) with tics was ~30 mg/day. In a larger RCT of older children & adolescents (13.6 y) with bipolar disorder, the mean dose was 120 mg/day in those weighing >45 kg.		
Special populations	Dosage adjustments are generally not required on the basis of gender, race, or renal function. Dose reduction of 40-50% is recommended in the presence of severe hepatic insufficiency and of 20-30% in adolescents, first episode psychosis, organic brain syndrome, bipolar maintenance, underweight, and Asian ethnicity.		
Potent inhibitors and inducers of CYP3A4	Dose modification of ziprasidone may be warranted. Pharmacokinetic studies demonstrate the potential for an estimated 30-40% change in systemic exposure to ziprasidone when co-administered with potent CYP3A4 inhibitors (e.g., clarithromycin, protease inhibitors, ketoconazole) and inducers (e.g., carbamazepine, St John’s wort).		

* All doses to be taken with a meal of at least 500 kcal to ensure adequate and consistent absorption.

A notable challenge to initiating ziprasidone therapy is its association with activation and insomnia early in the course. In general, slower, more cautious titrations have been associated with poorer outcomes. As such, this panel recommends a more rapid titration schedule to target doses and, when necessary, the limited-term use of anxiolytics to support treatment initiation (e.g., lorazepam 1–2 mg/day as needed × 7 days). Patients should be advised that they might experience some restlessness or agitation, which frequently subsides with dosage escalation and time or may benefit from a short course of a benzodiazepine. If the presentation is more fitting with a diagnosis of antipsychotic-induced akathisia, rapid dosage escalation is not recommended. Rather, anti-akathisic treatment such as propranolol can be considered and dosage escalation continued if akathisia subsides.

#### Switching

The method of switching between antipsychotics varies, however the most commonly applied method is the overlapping switch. In doing so, there are multiple factors to consider before embarking on a switch in antipsychotic therapy that involves ziprasidone [190-192]. These include the reason for the switch (e.g., lack of response, intolerance, non-adherence), the pharmacologies of the antipsychotics being stopped and started (e.g., high or low antagonist potency at dopamine, histamine, and muscarinic receptors), and dosing history (e.g., duration, daily dose).

Clinical experience suggests that higher doses of a replacement antipsychotic are usually needed when previous antipsychotics have proven inadequately effective and that lower doses are indicated with a history of intolerance to previous agents. These experience-based principles apply to dosing targets when switching to ziprasidone. Also, when the pharmacologies of the outgoing and incoming antipsychotic are at odds with each other (e.g., high vs. low potency at dopamine, histamine, or muscarinic receptors) a more careful switch is warranted. For example, when switching from olanzapine, quetiapine, or clozapine to ziprasidone, an excessively rapid transition may be associated with activation and signs and symptoms of cholinergic rebound due to the rapid loss of the antihistaminc and/or anticholinergic effects. Attention to sleep hygiene measures (e.g., avoid late-day use of caffeine and other stimulants, rise at the same time each day, etc.) and short-term use of sedatives can help address this when it is necessary to reduce the doses of these drugs quickly. D2 related adverse effects, such as EPS, may also worsen transiently due to excessive D2 blockade if down titration of the current antipsychotic is too slow. This latter issue is especially of concern when switching between risperidone, paliperidone, or other potent D2 antagonists with longer half-lives and ziprasidone.

## Conclusion

The available literature and discussions of clinical experience reviewed in this paper support the use of ziprasidone in schizophrenia and bipolar mania. There is less information available to support its use in other indications including depression and anxiety or for special groups including children, youth, and elderly. Ziprasidone’s main advantages over other antipsychotics include its attractive metabolic effects profile. Clinicians should prioritize ziprasidone for patients with pre-existing metabolic issues (e.g., obesity, dyslipidemia, impaired glucose tolerance), patients with metabolic adverse effects to other antipsychotics, and for patients wishing to mitigate the risk for metabolic adverse effects when considering their antipsychotic options. Patients should be informed that ziprasidone has a high rate of early treatment discontinuation, primarily due to a lack of efficacy and/or adverse effects, and that the risk for this is lower with some but not all other antipsychotics. A remarkable feature is that ziprasidone clearly needs to be taken with food, an issue that may underlie a proportion of treatment failures in trials and clinical practice. As with other antipsychotics, optimal dosing of ziprasidone has evolved with experience and new clinical information. To maximize ziprasidone’s benefits and limit early intolerance, we advocate targeting the high end of the dosing range of ziprasidone along with the limited term use of a benzodiazepine to manage the not uncommon experience of activation or agitation with treatment initiation.

## Abbreviations

BMI: Body mass index; CANMAT: Canadian Network for Mood and Anxiety Treatments; CATIE: Clinical Antipsychotic Trials of Intervention Effectiveness; CGI: Clinical Global Impression; ECG: Electrocardiogram; EPS: Extrapyramidal Symptoms; EUFEST: European First Episode Schizophrenia Trial; HAM-D: Hamilton Rating Scale for Depression; MADRS: Montgomery-Asberg Depression Rating Scale; MDD: Major Depressive Disorder; MRS: Mania Rating Scale; NPI: Neuropsychiatric Inventory; PANSS: Positive and Negative Syndrome Scale; QLS: Quality of Life Scale; SSRI: Selective Serotonin Reuptake Inhibitor; TdP: Torsades de pointes; WFSBP: World Federation of Societies of Biological Psychiatry.

## Competing interests

Dr. Andrea Murphy, Dr. Stan Kutcher, Dr. Carlo Carandang, and Dr. Jorge Soni declare that they have no competing interests.

Dr. David Gardner has been involved with the advisory board for Janssen-Ortho and has received education grant funding from Pfizer for this project.

Dr. Serge Beaulieu has been a speaker for Astra Zeneca, Biovail, Bristol Myers Squibb (BMS), Eli Lilly, GlaxoSmithKline (GSK), Janssen-Ortho, Lundbeck, Organon, Oryx, Pfizer, Wyeth. He has acted on the advisory board and/or as a consultant for Astra Zeneca, BMS, Eli Lilly, GSK, Janssen-Ortho, Lundbeck, Oryx, Otsuka, Schering-Plough Merck, Pfizer. He has also received research support from Astra Zeneca, Biovail, BMS, Eli Lilly, Janssen-Ortho, Lundbeck, Merck-Frosst, Novartis, Pfizer, and Servier.

Dr. Alain Labelle has been involved with advisory boards for Pfizer and has been a speaker for Pfizer.

Dr. Pierre Lalonde has been involved with advisory boards for BMS and Pfizer, and has also been a speaker for BMS and Pfizer.

Dr. Ashok Malla has received grant support from Astra Zeneca, Pfizer, BMS and Janssen Ortho. He has been involved with the advisory board and acted as speaker honoraria for Janssen, BMS, Pfizer, Astra-Zeneca, Sunovion, Lundbeck, and Wyeth.

Dr. Heather Milliken has been involved as a speaker, member of advisory board, clinical trals and/or educational grants for Janssen-Ortho, Lundbeck, Otsuka, Pfizer, Roche, Sunovion

Dr. Claire O’Donovan has received Research Funding from Elan Pharma, Pfizer, Cephalon, Brain Cells Inc. She has also been involved with the advisory boards for Astra Zeneca, Pfizer, BMS, Sunovion, Lundbeck, and Servier.

Dr. Ayal Schaffer has been involed with the advisory board and speaker’s bureau for and has received research grants from AstraZeneca, BMS, Canadian Network for Mood and Anxiety Treatments, Eli Lilly, Lundbeck, and Pfizer.

Dr. VH Taylor has been a speaker for Lilly, Pfizer, Lundbeck, BMS and Allergan and has received grant funding from BMS.

Dr. Richard Williams has received research, ad boards, and travel support from Pfizer, Janssen-Ortho, Roche, Mylan, Lundbeck, and BMS.

## Authors’ contributions

All fourteen authors were convened on April 16, 2010 in order to discuss and synthesize suggestions for the clinical use of ziprasidone based on current research and clinical experiences. DG and SK served as the co-chairs for the meeting and AM and DG led the development of the manuscript. Members were delegated a specific clinical area to critically analyze and present at the meeting. During the development of this manuscript, all members were provided with two opportunities for critical and constructive feedback. This manuscript, drafted by AM and DG, represents a synthesis of information from individual presentations, collective discussions, an extensive examination of the evidence base, and feedback from all panel members. All authors read and approved the final manuscript.
